# Pyrolysis Process of Mixed Microplastics Using TG-FTIR and TED-GC-MS

**DOI:** 10.3390/polym15010241

**Published:** 2023-01-03

**Authors:** Min-Hyun Cho, Yu-Jin Song, Chan-Joo Rhu, Byung-Rye Go

**Affiliations:** Korea Conformity Laboratories, Seoul 08503, Republic of Korea

**Keywords:** microplastics, pyrolysis, quantification analysis, TG-FTIR, TED-GC-MS

## Abstract

Microplastics have become a ubiquitous contaminant in the environment. The present study focuses on the identification, characterization, and quantification techniques for tracking microplastics. Due to their unique compositional structure, unambiguous identification of individual polymers in various plastic samples, usually comprised of mixtures of individual polymers, remains a challenge. Therefore, there is limited research on the pyrolysis characterization of mixed samples. In this study, two analytical methods, TG-FTIR and TED-GC-MS combined with thermogravimetric analysis were used to evaluate the thermal-degradation process of individual and mixed samples of polypropylene (PP), polyethylene terephthalate (PET), and polyvinyl chloride (PVC). The primary interaction was the volatilization of terephthalic acid bound to chlorine molecules. The reduction of vinyl-ester functional groups and aromatic hydrocarbon intermediates related to olefin branching was confirmed. Char formation was increased, due to aromatic compounds from PET and PVC. All of the polymers used in the study may be underestimated in quantity, due to combined volatilizations during pyrolysis. TG-FTIR and TED-GC-MS showed forceful advantages in identifying mixed microplastics through different discrimination mechanisms. The study provides deep insight into pyrolysis behaviors and the interactions of mixed polymers, and the obtained results can help better comprehend the complex pyrolysis process.

## 1. Introduction

Microplastic particles are garnering a great deal of attention as one of the most pressing environmental issues, due to today’s vast plastic consumption [1,2,3,4]. All types of plastic products, including cosmetics, abrasives, and pharmaceuticals, which are consumed in an inappropriate manner such as in human activities [5,6] and the use of tire and road wear particles (TRWP) [7], are known to be sources of excessively generated plastic pieces. The generated microplastic particles enter the environment via various channels and become ubiquitous in the air, soil, the underground and surface water, the oceans, and in marine life [8,9,10,11,12]. Through the food chain, organisms may be exposed to microplastics that are fragmented into even smaller pieces (less than 5 mm) through resource circulation [13], and a potential threat is imposed on humans as they move up the food chain [14,15]. Therefore, it is becoming more important to identify and manage contamination caused by microplastic behavior in the environmental matrices [16,17,18].

A reliable analysis method is required as a means of monitoring tin order to investigate and control the impacts of microplastics [19,20]. Studies on methodologies for identifying and quantifying microplastics have been continuously conducted for the last several years [21,22]. For microplastic quantification, spectral analytical methods using Fourier-transform infrared spectroscopy (FTIR) and Raman methods, and thermal analysis methods such as gas chromatography-mass spectrometry (GC-MS) and differential scanning calorimetry (DSC) are representative approaches [23,24]. Since each analytical tool has respective detection limits, strengths, and weaknesses in quantifying mixed microplastics [25], the identification and quantification of mixed microplastics is continuously assessed through the development of various analytical and pretreatment techniques [26,27,28]. Pyrolysis, in particular, is the most robust quantification process, as it can analyze the properties of compounds and the resulting pyrolytic products by analyzing the thermal characteristics of the samples [29]. FTIR and GC-MS integrated with thermogravimetric analysis (TGA) have been proposed as powerful tools to analyze the pyrolytic products of plastics. These two methods can specify the type of mixed microplastics, through different approaches. FTIR uses infrared rays to recognize certain functional groups or molecular motion to identify the molecule through vibrational energy, whereas GC-MS identifies and quantifies the ionized molecules by separating degraded compounds according to the mass-to-charge ratio. In this process, intermediates and quantitative ions are selected for individual polymers to identify specific polymers. However, this could hamper the quantification of mixed polymers, due to secondary reactions between polymers, and the interactions of the sample plastic mixture within the environmental matrices are not taken into account in the process [30]. Accordingly, there are growing attempts to quantify mixed microplastics based on existing studies on homopolymers [31,32,33]. However, it is difficult to establish a standardized analysis procedure, due to a variety of formulas and origins of polymers [34,35], and the identification of each polymer being hindered by interactions in the pyrolysis [36,37] and the altered kinetics [38,39] of mixed microplastics, in addition to the presence of persistent organic pollutants [40], makes it difficult to perform a precise quantitative analysis. Specifically, polyvinyl chloride (PVC), an amorphous polymer, is difficult to detect using GC-MS, due to the low specificity of pyrolysis products [41] and the presence of chlorine ions in molecules, and it is known as a substance that hinders identification using decomposition temperatures with other semi-crystalline polymers in thermal extraction-desorption gas chromatography/mass spectrometry (TED-GC-MS) [42] and TGA methods [43].

This study applied both TG-FTIR and TED-GC-MS as tools for a quantitative analysis of mixed microplastics including polypropylene (PP), polyethylene terephthalate (PET), and polyvinyl chloride (PVC), the polymers that are the most widely found in environmental matrices. Based on the pyrolysis results of each material, the interaction between polymers was observed during the pyrolysis process of mixed microplastics. By examining the differences between the two analytical tools for PVC (a polymer that has been extensively evaluated in previous studies), based on the interference during the analysis, this study will provide a useful reference for a valid comparison of each analytical technique and the optimization of analytical methodologies.

## 2. Materials and Methods

### 2.1. Materials

The microplastics, specifically polypropylene (PP), polyethylene terephthalate (PET), and polyvinyl chloride (PVC), used in this study were provided by the Korea Testing & Research Institute, Gwacheon, Korea (KTR) as pure substances for standard analysis. The data of the plastic materials are presented in Table S1. The ultimate analysis of the plastics was conducted by an elemental analyzer (FlashSmart, Thermo Fisher Scientific, Massachusetts, US). The mixtures of microplastics (MP) used in the analysis were prepared by mixing single types of polymers in a weight ratio of 1:1:1 (PP, PET and PVC).

### 2.2. TGA-FTIR

TGA-FTIR was conducted on a TGA4000 (Perkin Elmer, Massachusetts, US) combined with Spectrum 3 (Perkin Elmer, Massachusetts, US). The sample, loaded into a ceramic crucible, was heated at a rate of 20 K/min from 303 to 973 K with a nitrogen flow rate of 100 mL/min. The pyrolysis gas moved through the transfer line to the detection unit, and the temperature of the transfer line and FTIR gas cell was maintained at 543 K, to prevent condensation of the pyrolysis gas. FTIR spectra were measured using an MCT detector (TL8000, Perkin Elmer, Massachusetts, US) (10,000–450 cm^−1^) with a range of 4000–600 cm^−1^ at a spectral resolution of 16 cm^−1^. Each analysis was conducted more than three times under the same experimental conditions.

### 2.3. TED-GC-MS

The samples of all plastic reference materials used for the analysis were placed in 150 μL aluminum oxide crucibles, and TGA was performed on a TGA 2 (Mettler/Toledo, Gieβen, Germany) equipped with an autosampler and horizontal thermobalance, at a heating rate of 10 K/min from 323 to 973 K, with a nitrogen flow rate of 100 mL/min. The pyrolysis gas was adsorbed to the polydimethylsiloxane (PDMS) stir bar sorptive extraction (SBSE) Twister^®^ (GERSTEL, Mülheim an der Ruhr, Germany) in a thermal desorption unit, TDU2 (GERSTEL, Mülheim an der Ruhr, Germany), and the temperature of the connected TDU was maintained at 513 K, to prevent gas coagulation. The adsorbed Twister^®^ was moved through a multipurpose sampler, MPS (GERSTEL, Mülheim an der Ruhr, Germany), to the TDU coupled to GC (8890, Agilent Technologies, Palo Alto, CA, USA). After performing cryo-focusing through the process of heating after cooling the Twister^®^ to 173 K with the cooled injection system (CIS) in the TDU, thermal desorption was conducted. The MS Detector 5977B (Agilent Technologies, Palo Alto, CA, USA) was operated in a range of 35–350 m/z in the electron-impact mode (EI) at 70.0 eV. Each experiment was repeated three times or more under the same conditions, and the Twister^®^ was disposed of after a single use, to prevent environmental errors. Detailed GC-MS conditions are shown in Table S2. The qualitative identification of pyrolysis products was performed using electron-impact mass-spectral libraries such as F-Search polymer analysis software (Frontier Lab, Fukushima, Japan) and NIST17 Library (National Institute of Standards and Technology: Gaithersburg, MD, USA). The results of the extracted-ion chromatogram (EIC) spectrum were used as references for targeting specific ions, in an effort to clearly identify the compounds and simultaneously run a full spectrum scan on the SCAN mode.

## 3. Results and Discussion

### 3.1. Results of Pyrolysis through Each Instrument

**Figure 1:**
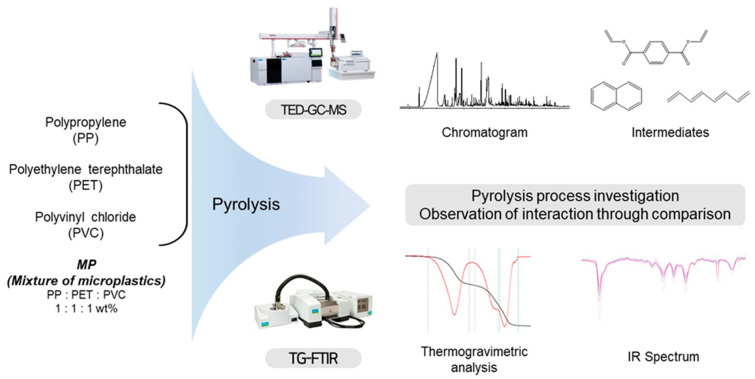
Schematic diagram of comparative experimental methods of two instruments to investigate the pyrolysis process of microplastics (PP, PET, PVC and mixture of polymer).

Two pyrolysis analysis tools were used together to identify the thermal decomposition characteristics of microplastics and to observe and evaluate the thermal-degradation stages, intermediates, and interactions of the polymer. A schematic diagram of the experiment including the factors evaluated in each method is presented in Figure 1.

**Figure 2:**
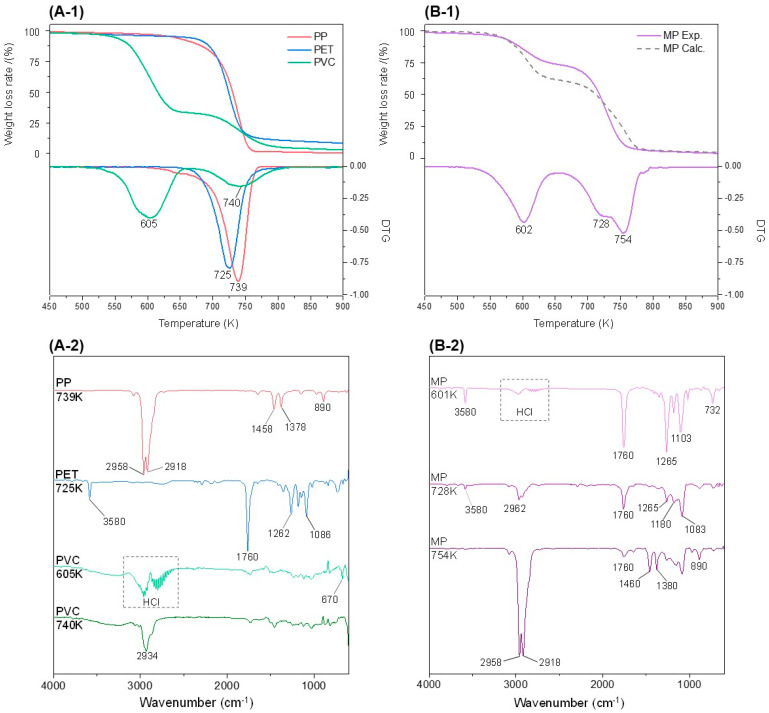
(**A-1**) TG, DTG curves for pyrolysis of PP, PET, PVC; (**A**-**2**) independent FTIR spectra of PP, PET, and PVC; (**B-1**) TG, DTG curves of MP; (**B-2**) FTIR spectra for each temperature peak of MP.

Figure 2(A-1) shows the curves for mass loss (degradation) and derivative thermogravimetry (DTG) with temperature during the pyrolysis of a single microplastic (PP, PET, PVC) under a nitrogen atmosphere. The key FTIR spectrum curves for the transferred pyrolysis gas were selected in the maximum absorbance section for each pyrolysis stage, and are shown in Figure 2(A-2). PP and PET showed one T_max_ (temperature of maximum weight-loss rate) in a single stage, and two-stage pyrolysis took place for PVC. The ranges of the pyrolysis reaction stages of the single polymer and MP, as well as the obtained T_max_, are summarized in Table S3. For PP, weight loss following pyrolysis was observed from a temperature of 630 K but at a slow decomposition rate, and then the pace of pyrolysis was accelerated at 700 K or higher. Pyrolysis ended at 770 K with almost no residue generated (>0.01%). At sharp peaks of 2958 cm^−1^ and 2918 cm^−1^, indicating the presence of alkyl groups including -CH_3-_ and -CH_2_-, C-C bonds are randomly broken down to produce alkenes [44], and at peaks of 1458 cm^−1^ and 890 cm^−1^, the C=C bonds of the alkenes that have been cut can be found [45]. The peak of 1378 cm^−1^ exhibits C-H bending, which indicates that the sample is PP plastic [46]. PET can be easily converted to aromatic hydrocarbons and degradation products containing a C=O stretching bond at 1260 cm^−1^ through deoxygenation reactions of O-H stretching, mainly originating from its own oxygen atoms at 3583 cm^−1^ and benzoate carboxylic acid at 1760 cm^−1^. The low peak-intensity of 2400–2200 cm^−1^ indicates that a small amount of CO and CO_2_ is produced during the pyrolysis of PET [47]. As for single pyrolysis, in which deoxygenation of the carboxyl group is not assisted, a large amount of char is generated, due to the high content of poly-aromatics [48]. During the pyrolysis of PVC, the first reaction took place after HCl was removed by the cleavage of the C-Cl bond at 605 K, and then the pyrolysis products containing CH_2_ bending of 2934 cm^−1^ appeared in the second stage. The distribution of complex spectra near 1500 cm^−1^ suggests that aromatic degradation compounds were detected [49]. The weight loss in the first-stage reaction of PVC was approximately 66%, and then reached 96% in the second-stage reaction. After the pyrolysis process ended, approximately 6.8 and 1.8 wt% of black residues were generated for PET and PVC, respectively.

Figure 2(B-1) shows the results of a TG and DTG analysis of MP. The average of the single-polymer degradation values was calculated and used as a control group (MP Calc.). The pyrolysis step corresponding to the first-stage degradation reaction of PVC was completed in the 520–650 K range, a range narrower than the 460–650 K observed during the pyrolysis of a single polymer. The end-temperature of the first stage of thermal degradation was 650 K for both PVC and MP; however, roughly 38.8% of weight loss was observed when the first stage of thermal degradation ended. This amount of loss is approximately 11.3% higher than 27.5%, the average weight loss of PP, PET, and PVC at the same stage of the reaction, showing that the corresponding thermal degradation reaction may have accelerated, due to interactions. After the pyrolysis was completed, the amount of residual char generated in the MP was approximately 4 wt%, which was higher than that of the pyrolysis of single PVC and PET, due to a greater formation of poly-aromatics and the intermediates of PP and PVC not assisting the deoxygenation of the PET pyrolysis products.

The FTIR spectra for each temperature peak of MP are shown in Figure 2(B-2). The phenols formed by the HCl separation of the PVC that can be identified at 670 cm^−1^ assist the formation of C=O carbonyl groups, through interactions with O-H out-of-plane bending from 520 K [50]. The carbonyl group indicated by the strong peaks at 1760 cm^−1^ and 1265 cm^−1^, formed from a temperature lower than the temperature of the pyrolysis of single PET, showed a gradually declining trend after reaching the highest peak-intensity at 601 K, exhibiting a synergistic effect of the pyrolysis between PET and PVC. The C-O stretching peak at 1103 cm^−1^ indicated that aliphatic ether groups were formed with the above interactions. The low peak-intensity of O-H stretching at 3580 cm^−1^ at 670 K showed that the dehydrogenation within the mixed sample was almost ended. A minute formation of CO and CO_2_, stemming from the oxygen atoms of PET, was found at 2400–2200 cm^−1^. It was discovered that the degradation of synthesized alkenes took place most actively at 728 K, based on the stretch peak for CH_n_ at 2958 cm^−1^ and 2918 cm^−1^, the C-H bending at 1460 cm^−1^ and 1380 cm^−1^, and the peak at 890 cm^−1^.

As a result of comparing the actual weight loss in the pyrolysis of the MP with the weight loss calculated by the TGA analysis of single polymers, during the degradation of the alkenes from PP, PP and PVC acting as hydrogen donors intervened in the synthesis of alkanes and alkenes with more aromatic ester-based intermediates at 700–730 K, due to the aromatic hydrocarbons of PVC and PET. This in turn shifted the pyrolysis temperature of alkane and alkene to higher temperatures, and increased the amount of char generated.

Single TED-GC-MS chromatograms of PP, PET, and PVC are shown in Figures S1–S3, and the thermal-degradation compounds of each polymer identified through the spectra analysis are summarized in Tables S4–S6. PP was mostly degraded into aliphatic hydrocarbons of alkenes and alkadienes. It was broken down into intermediates of various size, due to the random cleavages of C-C in the polymer chain of polypropylene, forming compounds (m/z: 69, 70) containing carbon double bonds resulting from the hydrogen transfer due to C-H cleavage. This resulted in the formation of alkenes and dienes in the PP pyrolysis, which is consistent with the previously proposed mechanism [51]. Aromatic compounds containing carboxyl groups and methyl groups were mainly detected as degradation products of PET. Other degradation products, in addition to the aromatic compounds, included divinyl terephthalate (m/z: 175, 104, 76), vinyl benzoate (m/z: 105, 77, 51), and benzoic acid (m/z: 122, 105, 77) derived from monomers, and biphenyl (m/z: 154, 153, 152) and anthracene (m/z: 178, 179, 176), which consist of benzene. The results of the GC-MS analysis of PVC identified several polycyclic hydrocarbons and benzenoid aromatic compounds that were not detected clearly in the FTIR spectrum.

**Figure 3:**
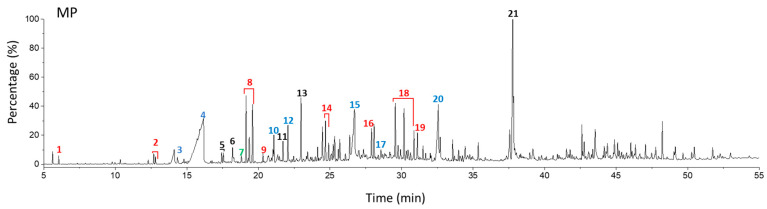
TED-GC-MS chromatogram of MP. The color of the number indicates the origin: red: PP, blue: PET, green: PVC, black: compounds newly appearing in MP.

Figure 3 shows the TED-GC-MS chromatogram of the MP, and the identified compounds are listed in Table 1. 1,4-benzenedicarboxylic acid and t2-chloroethyl ester (MP No. 20), chlorinated derivatives of benzoic acid and terephthalic acid, which were reported to be formed by the interaction between PET and PVC [52], showed the highest peak. It was discovered that a considerable number of PET monomers had been substituted with benzoic acid or chloroethyl benzoate and chlorinated terephthalic acid, based on the m/z = 211, 213 and the ion ratio of 3:1, due to chlorine ions. Peaks identified to be 2-chloroethyl benzoate, terephthalic acid, di(2-chloroethyl) ester, and adipate (m/z: 129, 171, 111) were newly found in the thermal degradation of MP. The share of ester-based compounds that could be identified at various RTs increased, while the amounts of naphthalene, biphenyl, and terephthalic-acid generation, decreased.

### 3.2. Pyrolysis Process

#### 3.2.1. Pyrolysis Process of PP

**Figure 4:**
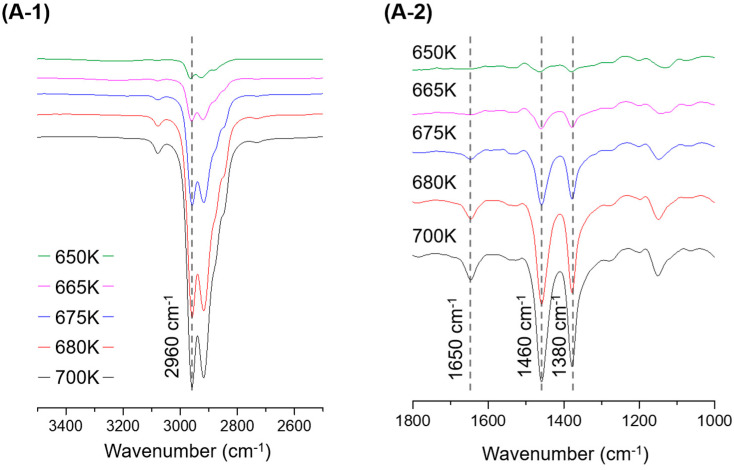
IR Spectrum drift at the end of PP pyrolysis. (**A-1**) 3400–2500 cm^−1^, in the range of 650–700 K; (**A-2**) 1800–1000 cm^−1^, in the range of 650–700 K.

During the PP pyrolysis reaction, alkanes, alkenes, and alkadienes of widely distributed carbon numbers, including the methyl groups randomly cut in the main chain, were identified. The single curve at 1650 cm^−1^ of conjugated C=C indicated that the pyrolysis products of PP were mainly alkenes. The shortening of the vibration band of C-H at 2960 cm^−1^ and 2920 cm^−1^ after T_max_ temp, and the constant decrease in the CH_2_ methylene group at 1460 cm^−1^ and CH_3_ methylene group at 1380 cm^−1^, which can be seen in Figure 4, indicated that the pyrolysis products of PP were formed at a constant rate, irrespective of change in temperature. The pyrolysis reaction pathway of PP is presented in Figure S4.

#### 3.2.2. Pyrolysis Process of PET

**Figure 5:**
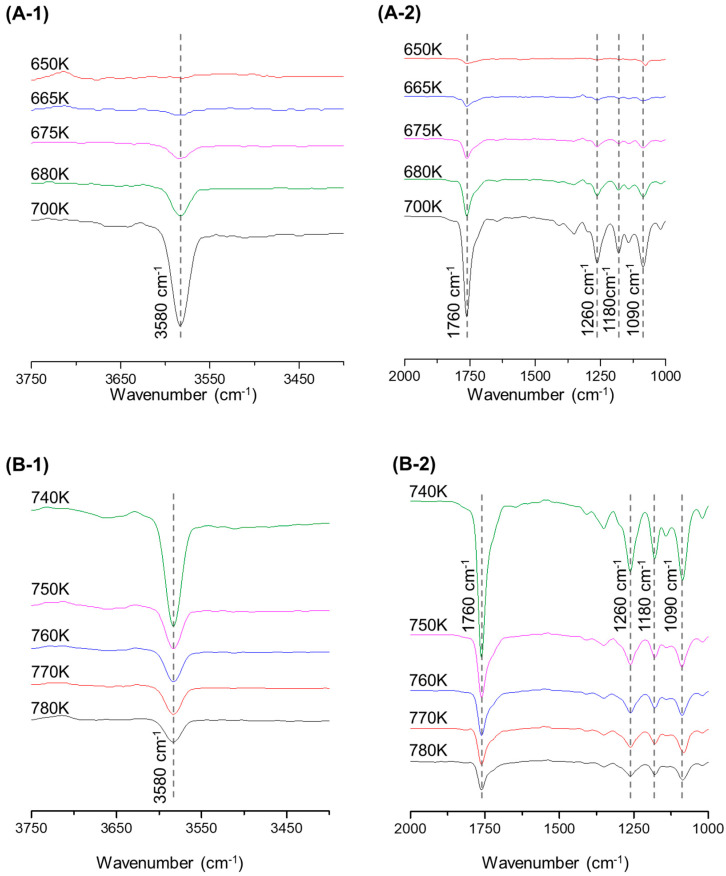
TG-FTIR spectrum drift by temperature during PET pyrolysis: (**A-1**) 3750–3400 cm^−1^, in the range of 650–700 K; (**A-2**) 2000–1000 cm^−1^, in the range of 650–700 K; (**B-1**) 3750–3400 cm^−1^, in the range of 740–780 K; (**B-2**) 2000–1000 cm^−1^, in the range of 740–780 K.

Figure S5 represents the pyrolysis reaction path of PET with the IR spectrum and GC-MS data taken into account. In the early stage of the pyrolysis of PET, the ester bonds of the main chain are cleaved and broken down into molecules containing a benzene-carboxyl group and vinyl end groups such as 4-((vinyloxy)carbonyl)benzoic acid and 4-((2-hydroxyethoxy)carbonyl)benzoic acid, due to the six-membered cyclic-transition-state reaction. The first observed C=O at 1750 cm^−1^ and C-H at 1090 cm^−1^ spectra at 665 K, as shown in Figure 5(A-2), indicated that C-O cleavage of the vinyl end group led to the formation of acetaldehyde. Thereafter, the peaks at 1760 cm^−^^1^ and 1260 cm^−1^ grew sharply. As the peaks at 1760 cm^−1^ and 1090 cm^−1^, in which carboxyl groups and aromatic C-H out-of-plane bend bonds appear, grew, molecules broken down into benzoic acid, vinylbenzoic acid, methylbenzoic acid, and small amounts of phthalate acid, were identified. The peak of the C-O 2000 cm^−1^ range was observed near 725 K, showing a possibility of the release of CO and CO_2_ in the pyrolysis step, to form benzoic acid [53]. The O-H bonds and carboxyl groups with C-H in-plane bending vibration were detected at 3580 cm^−1^ and 1760 cm^−1^, respectively, with decreasing intensity until the end of the pyrolysis of PET; the intensity at 1180 cm^−1^ and 1090 cm^−1^ remained unchanged, as shown in Figure 5(B-1,B-2), consistent with the GC-MS analysis results, where aromatic compounds such as 4-methylbenzoic acid, 2-methylbiphenyl, and anthracene were detected. The formation of PAH in the pyrolysis of PET signals that PAH can also be formed through some radical reaction at a pyrolysis temperature of 950 K and higher [54].

#### 3.2.3. Pyrolysis Process of PVC

The C-Cl bond was cleaved first, and vaporized in the form of HCl. The radical movement led by the cleavage of C-Cl can form C=C allyl groups in the PVC main chain, and is converted into conjugated polyenes, including alkenes and dienes. The benzene derivatives, whose existence can be identified by GC-MS, were not identified by the distribution of bonds on the IR spectrum that helped determine the second stage of pyrolysis of PVC. Therefore, the thermal degradation pathway of PVC suggested in Figure S6 incorporated the highly probable assumptions regarding cycloaddition and condensation, which were consistent with the previous studies on the formation of benzene derivatives and PAHs among the pyrolysis products obtained from the analytical data [55,56,57,58,59]. The pyrolysis products include HCl, chlorinated hydrocarbons, chlorinated aromatic hydrocarbons, PAHs including naphthalene, and cleaved alkanes of small molecular weight.

#### 3.2.4. Pyrolysis Process of MP

**Figure 6:**
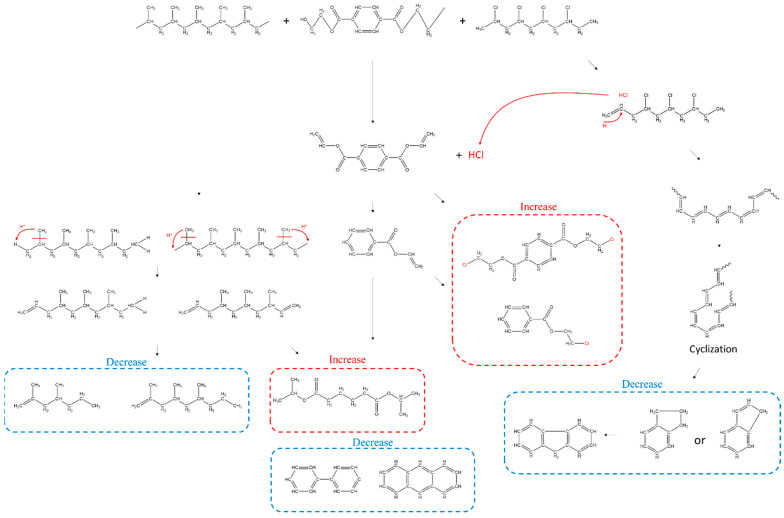
Overview of the occurrence of some pyrolytic compounds inferred from the pyrolysis process of MP.

**Figure 7:**
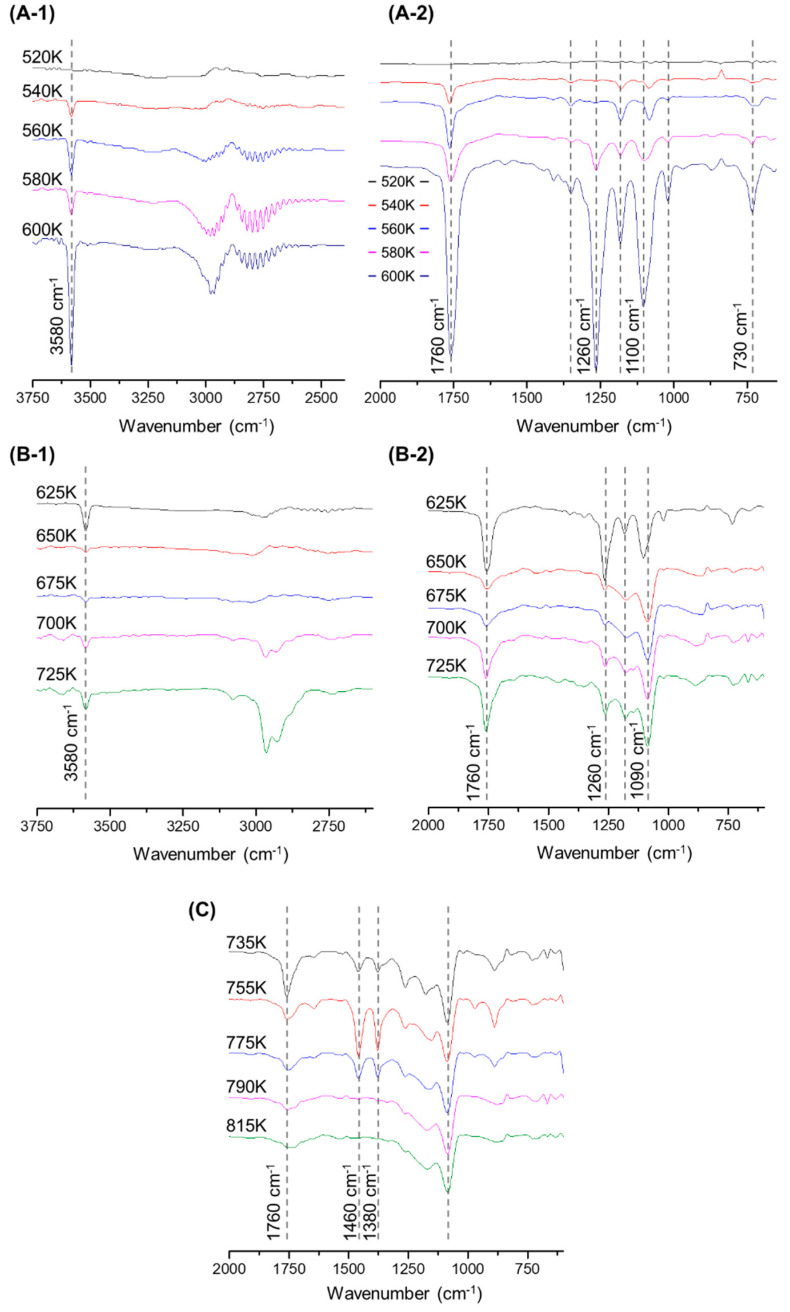
TG-FTIR spectrum drift by temperature during MP pyrolysis: (**A-1**) 3750–2400 cm^−1^, in the range of 520–600 K; (**A-2**) 2000–700 cm^−1^, in the range of 520–600 K; (**B-1**) 3750–2400 cm^−1^, in the range of 625–725 K; (**B-2**) 2000–700 cm^−1^, in the range of 625–725 K; (**C**) 2000–700 cm^−1^, in the range of 735–815 K.

Comparing the pyrolysis of MP with a series of pyrolysis of a single polymer, the process with interactions and intermediates without interactions were examined and listed in Figure 6. Furthermore, the IR spectrum drift that can identify the binding of pyrolysis products with a change in temperature of the pyrolysis, is shown in Figure 7. The 3580 cm^−1^ O-H bend in Figure 7 emerged more strongly across the pyrolysis of MP (600–754 K) compared to the single pyrolysis, and the HCl bends caused by Cl degradation were particularly concentrated for a short time (560–620 K) in the pyrolysis of MP. The pyrolysis products of the MP generated at 600 K indicated benzene and C-Cl stretching at 732 cm^−1^ [31], implying that intermediates such as divinyl terephthalate and benzoic acids were replaced by chlorobenzoic acid and chlorinated terephthalic acid, as shown in (A2). The interaction of MP accelerated the thermal degradation reaction of PP. Compared to the pyrolysis of single PP, the C-H bond of 1460 cm^−1^ and 1380 cm^−1^ began to appear from 735 K, rather than 665 K for the single pyrolysis, and disappeared at 790 K, a temperature lower than that (950 K) of the single pyrolysis. As stated in the TGA analysis, the PP acting as a hydrogen donor in the degradation of intermediates, including an ester at 700–730 K, led to a lower formation of alkenes, and these alkenes, which formed less than in the single pyrolysis, were considered to be intensively degraded at 735–790 K. After the end of the carboxyl group reaction, as seen from the peaks at 1760 cm^−1^ and 1260 cm^−1^ going down and up, the initiation of the thermal degradation of alkenes and dienes was determined, as shown in Figure 7C. The C=O stretch represented by the peak at 1760 cm^−1^ strongly emerged throughout the pyrolysis of MP, exhibiting how carboxylic acids and carboxylic derivatives were continuously identified during the degradation process.

## 4. Conclusions

The pyrolysis process of single and mixed polymers was suggested, based on the TGA-FTIR and TED-GC-MS results, and the characteristics and formation rate of intermediates that appeared as a result of the interactions of the polymers were evaluated.

The PP and PVC acting as hydrogen donors in the pyrolysis of MP were mainly degraded at 730–790 K by the aromatic hydrocarbons derived from PET and PVC, facilitating the synthesis of aromatic ester-based intermediates. Through these reactions, the alkanes and alkenes were generated less, while the amount of char generation was increased. Thus, the quantification of PP and PET using 2,4,6,8-Tetramethyl-1-undecene and vinyl benzoate in MP has the potential to underestimate the amount of polymer. Cl radicals generated by the cleavage of C-Cl in the pyrolysis of MP can rapidly bind with phenyl radicals and PET intermediates, after the cleavage of acetaldehyde of PET, to form chlorobenzoic acid and chlorinated terephthalic acid at 600 K, and lower the proportions of biphenyl, methylbenzoic acid, and divinyl terephthalate. This can hinder the quantification of PET in the pyrolysis of the polymer. It is difficult to identify naphthalene, due to the overlapping of RT with benzoic acid, and methylnaphthalene can be used as a marker for PVC in MP.

This study is worth highlighting, because it offers the potential use of a variety of pyrolysis products categorized by cross-checking, in the identification and quantification of individual polymers in mixed microplastics.

## Figures and Tables

**Table 1:** Thermal-degradation compounds of MP shown in Figure 3.

No.	RT/Min	Compound	Structure	Formula	m/z
1	6.050	2,4-Dimethyl-1-heptene	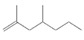	C_9_H_18_	70 43 55
2	12.686 12.810	2,4,6-Trimethyl-1-nonene	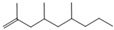	C_12_H_24_	69 57 43
3	14.343	Vinyl benzoate	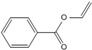	C_9_H_8_O_2_	105 77 51
4	16.175	Benzoic acid	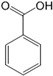	C_7_H_6_O_2_	122 105 77
5	17.429 17.550	Hydroxypropyl cellulose	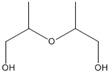	C_6_H_14_O_3_	59 103 57
6	18.192	1-Tridecene	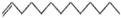	C_13_H_26_	70 55 83
7	18.810	2-Methylnaphthalene	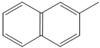	C_11_H_10_	142 141 115
8	19.137 19.357 19.587	2,4,6,8-Tetramethyl-1-undecene	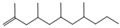	C_15_H_30_	69 111 85
9	20.320	2,4,6,8,10-Pentamethyl-1-undecene	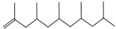	C_16_H_32_	69 83 57
10	21.088	Biphenyl	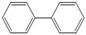	C_12_H_10_	154 153 152
11	21.724	2-Chloroethyl benzoate	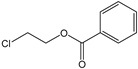	C_9_H_9_ClO_2_	105 122 77
12	22.059	2-Hydroxyethyl benzoate	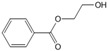	C_9_H_10_O_3_	105 123 77
13	22.978	Diisopropyl adipate	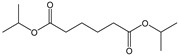	C_12_H_22_O_4_	129 171 111
14	24.682 24.902	2,4,6,8,10,12-Hexamethyl-1-pentadecene	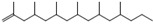	C_21_H_42_	69 111 71
15	26.713	Vinyl terephthalate	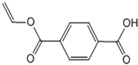	C_10_H_8_O_4_	149 121 65
16	27.905	2,4,6-Trimethyl-1-nonene	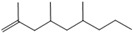	C_12_H_24_	71 57 84
17	27.348	Terephthalic acid	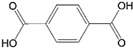	C_8_H_6_O_4_	149 166 121
18	29.564 30.174 30.871	2,4,6,8,10,12- Hexamethyl-1- pentadecene	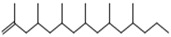	C_21_H_42_	69 111 83
19	31.110	2,4,6,8,10,12,14-Heptamethyl-1,14-pentadecadiene	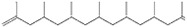	C_22_H_42_	69 83 109
20	32.690	Terephthalic acid	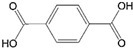	C_8_H_6_O_4_	149 166 121
21	37.767	Terephthalic acid, di(2-chloroethyl) ester	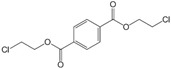	C_12_H_12_C_l2_O_4_	166 211 213

## Data Availability

The data presented in this study are available on request from the corresponding author.

## Supplementary Materials

The following supporting information can be downloaded at: https://www.mdpi.com/article/10.3390/polym15010241/s1, Figure S1: Single TED-GC-MS chromatogram of PP; Figure S2: Single TED-GC-MS chromatogram of PET; Figure S3: Single TED-GC-MS chromatogram of PVC; Figure S4: Partial pyrolysis pathway of PP proposed following the IR spectrum and TED-GC-MS; Figure S5: Pyrolysis pathway of PP proposed following the IR spectrum and TED-GC-MS, formation of some polycyclic aromatic hydrocarbons (PAH) may not be suggested by the IR spectrum and TED-GC-MS; Figure S6: The thermal decomposition pathway of PVC suggested by IR spectrum and TED-GC-MS results; Table S1: Material information, ultimate analysis of microplastics; Table S2: GC-MS instrumental parameter; Table S3: Thermogravimetric stage and Tmax for single polymer and MP; Table S4: Thermal degradation compounds of PP shown in Figure S1; Table S5: Thermal degradation compounds of PET shown in Figure S2; Table S6: Thermal degradation compounds of PVC shown in Figure S3.
