# Supplementary material for: Pyrolysis Process of Mixed Microplastics Using TG-FTIR and TED-GC-MS
Source: Polymers (Basel). 2023 Jan 3;15(1):241. doi: 10.3390/polym15010241 (PMC9824846; doi:10.3390/polym15010241)
Supplement: Supplementary file 1 [file polymers-15-00241-s001.zip › Supplementary material (Minhyun Cho)_ver.AP.pdf]

Supplementary material

Pyrolysis process of mixed microplastics using TG-FTIR and TED-GC-MS

Minhyun Cho[a], Yujin Song[a], Chan Joo Rhu[a], Byungrye Go[a]*

[a]Korea Conformity Laboratories. 199, Gasan digital 1-ro, Seoul 08503, South Korea

*Corresponding author.

E-mail address: innate@kcl.re.kr

Table S1. Material information, ultimate analysis of microplastics

Sample	Ultimate analysis (wt.%)					
	C	H	O	N	S	Cl
PP	85.08	13.98	-	-	-	
PET	62.58	4.31	33.12	-	-	
PVC	39.27	5.02	0.68	-	-	55.03

Table S2. GC-MS instrumental parameter

Parameter	Set value
CIS ramp rate	12K/s
CIS final temperature, hold time	543K, 5min
CIS split	20:1
GC initial temperature	313K
GC He flow rate	1ml/min
GC temperature ramp	5K/min
GC final temperature, hold time	573K, 5min
GC column	HP-5MS (Agilent J&W)
MS ion source temperature	503K
MS Quad temperature	423K

Table S3. Thermogravimetric stage and Tmax for single polymer and MP

	PP	PET	PVC	MP
1st stage Temp (K)	630-770	660-775	460-650	520-650
T_{max}	739	725	605	601
2nd stage Temp (K)	-	-	670-800	670-735, 735-795
T_{max}			740	728, 754

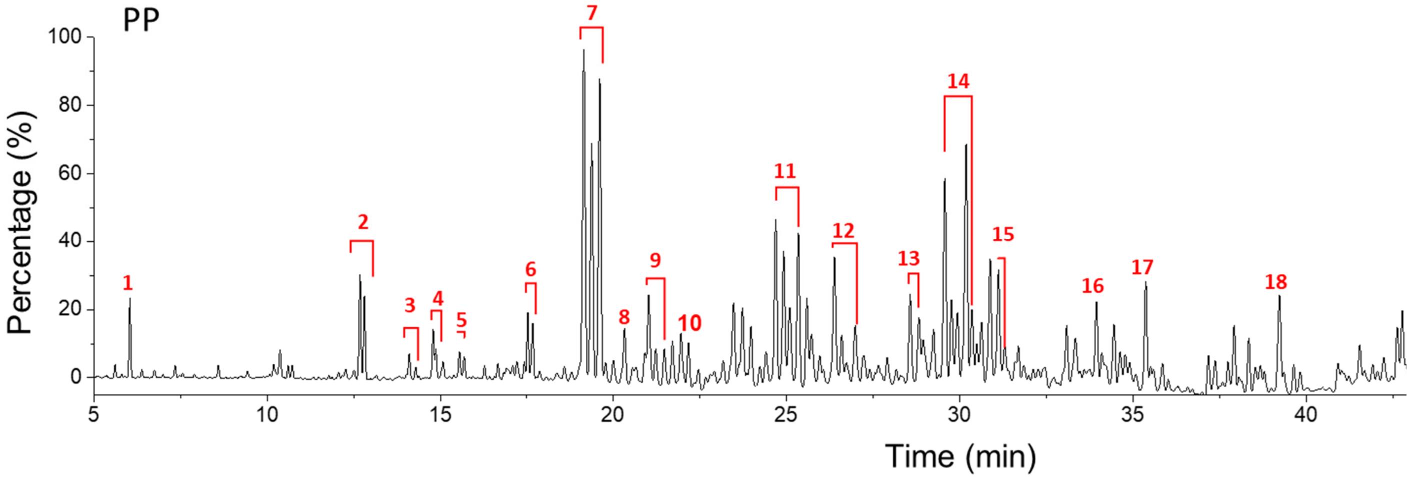

Figure S1. Single TED-GC-MS chromatogram of PP

Table S4. Thermal degradation compounds of PP shown in Figure S1

No.	RT/min	Compound	Structure	Formula
PP 1	6.044	2,4-Dimethyl-1-heptene	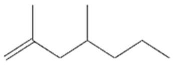	C_9H_{18}
PP 2	12.691<br>12.816	2,4,6-Trimethyl-1-nonene	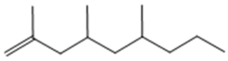	$C_{12}H_{24}$
PP 3	14.104<br>14.289	2,4,6,8,-Tetramethyl-1-nonene	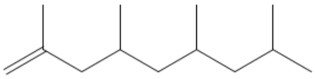	$C_{13}H_{26}$
PP 4	14.794<br>14.876	2,4,6,8-Tetramethyl-1,8-nonadiene	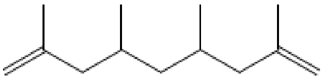	$C_{13}H_{24}$
PP 5	15.555<br>15.691	2,4,6,8-Tetramethyl-1-undecene	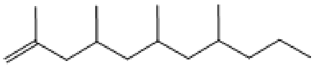	$C_{15}H_{30}$

PP 6	17.528<br>17.674	2,4,6,8,10,12-Hexamethyl-1,12-tridecadiene	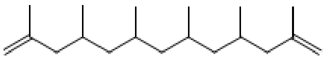	$C_{19}H_{36}$
PP 7	19.154<br>19.375<br>19.613	2,4,6,8-Tetramethyl-1-undecene	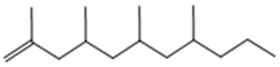	$C_{15}H_{30}$
PP 8	20.319	2,4,6,8,10-Pentamethyl-1-undecene	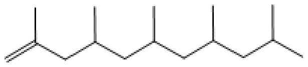	$C_{16}H_{32}$
PP 9	21.017<br>21.220<br>21.467	2,4,6,8,10-Pentamethyl-1,10-undecadiene	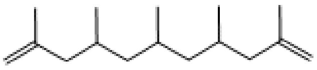	$C_{16}H_{30}$
PP 10	22.165	2,4,6,8-Tetramethyl-1-undecene	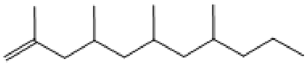	$C_{15}H_{30}$
PP 11	23.471<br>23.728<br>23.975	2,4,6,8,10,12,14-Heptamethyl-1,14-pentadecadiene	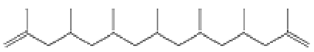	$C_{22}H_{42}$

PP 12	26.385<br>26.588<br>26.986	2,4,6,8,10,12-<br>Hexamethyl-1,12-<br>tridecadiene	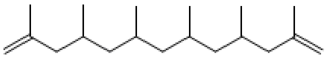	$C_{19}H_{36}$
PP 13	28.566<br>28.822	2,4,6,8,10,12,14-<br>Heptamethyl-1,14-<br>pentadecadiene	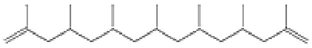	$C_{22}H_{42}$
PP 14	29.564<br>29.758<br>29.935<br>30.182<br>30.341	2,4,6,8,10,12-<br>Hexamethyl-1-<br>pentadecene	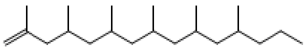	$C_{21}H_{42}$
PP 15	31.118<br>31.286	2,4,6,8,10,12,14-<br>Heptamethyl-1,14-<br>pentadecadiene	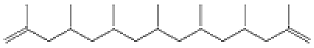	$C_{22}H_{42}$
PP 16	33.943	2,4,6,8,10,12,14,16,<br>18-nonamethyl-1-<br>heneicosene	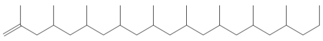	$C_{30}H_{60}$
PP 17	35.374	2,4,6,8,10,12,14,16,1<br>8-Nonamethyl-1,18-<br>nonadecadiene	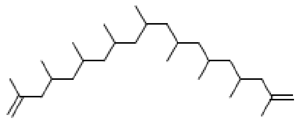	$C_{28}H_{54}$

PP 18	39.232	2,4,6,8,10,12,14,16,<br>18,20,22,24,26-<br>Tridecamethyl-1,26-<br>heptacosadiene	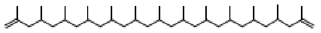	$C_{40}H_{78}$

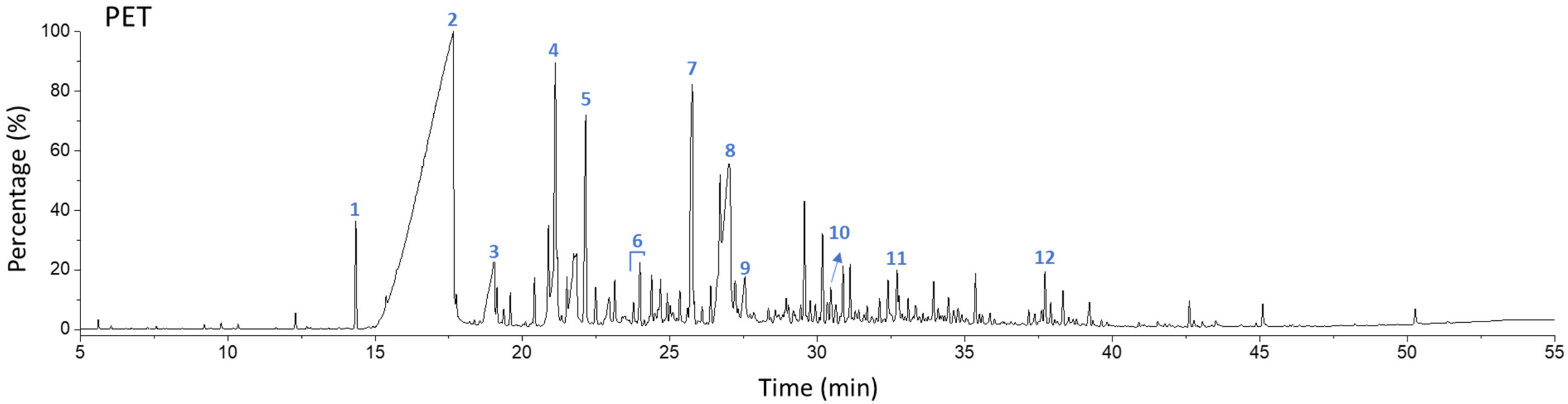

Figure S2. Single TED-GC-MS chromatogram of PET

Table S5. Thermal degradation compounds of PET shown in Figure S2

No.	RT/min	Compound	Structure	Formula
PET 1	14.346	Vinyl benzoate	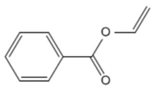	$C_9H_8O_2$
PET 2	17.667	Benzoic acid	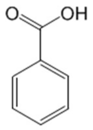	$C_7H_6O_2$
PET 3	19.032	4-Methylbenzoic acid	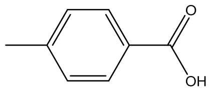	$C_8H_8O_2$
PET 4	21.120	Biphenyl	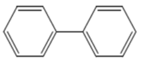	$C_{12}H_{10}$
PET 5	22.151	2-Hydroxyethyl benzoate	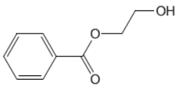	$C_9H_{10}O_3$
PET 6	23.771<br>23.983	2-Methylbiphenyl	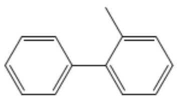	$C_{13}H_{12}$

PET 7	25.762	Divinyl terephthalate	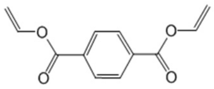	$C_{12}H_{10}O_4$
PET 8	26.996	Vinyl terephthalate	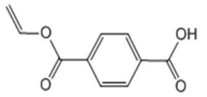	$C_{10}H_8O_4$
PET 9	27.550	Terephthalic acid	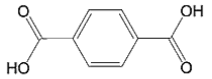	$C_8H_6O_4$
PET 10	30.457	Anthracene	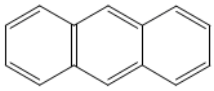	$C_{14}H_{10}$
PET 11	32.704	Biphenyl-4-carboxylic acid	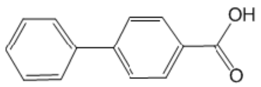	$C_{13}H_{10}O_2$
PET 12	37.707	Ethylene glycol dibenzoate	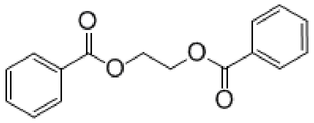	$C_{16}H_{14}O_4$

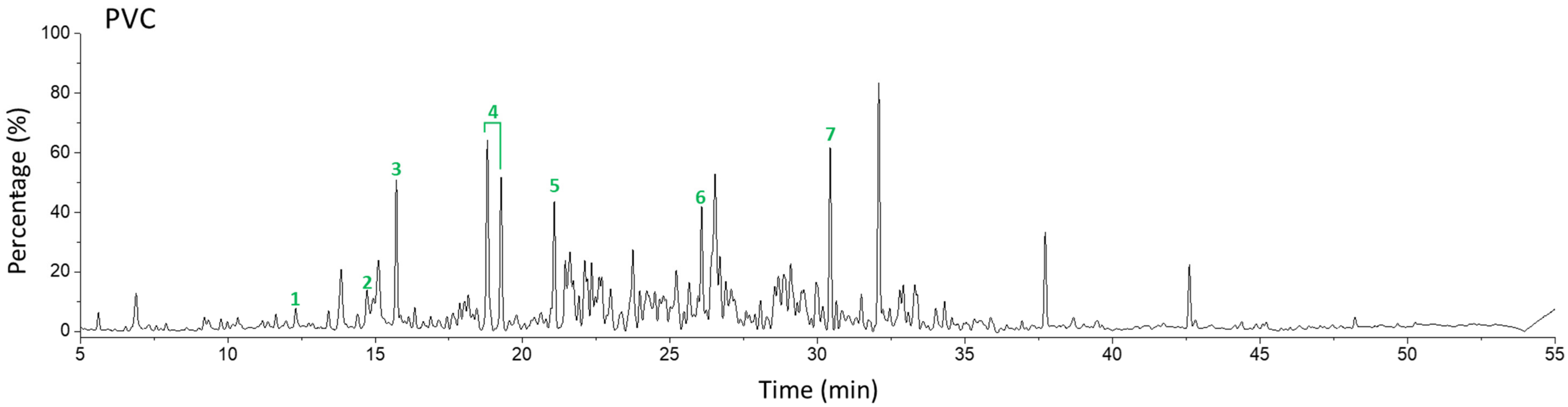

Figure S3. Single TED-GC-MS chromatogram of PVC

Table. S6. Thermal degradation compounds of PVC shown in FigureS3

No.	RT/min	Compound	Structure	Formula
PVC 1	11.636	Indene	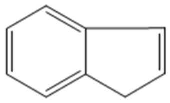	C_9H_8
PVC 2	14.715	1-Methylindene	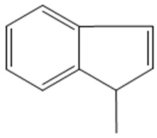	$C_{10}H_{10}$
PVC 3	15.717	Naphthalene	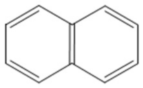	$C_{10}H_8$
PVC 4	18.807<br>19.283	2-Methyl<br>naphthalene	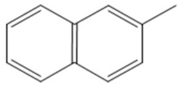	$C_{11}H_{10}$
PVC 5	21.070	Acenaphthene	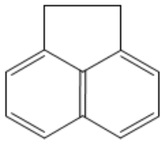	$C_{12}H_{10}$
PVC 6	26.069	Fluorene	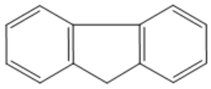	$C_{13}H_{10}$
PVC 7	30.447	Anthracene	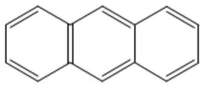	$C_{14}H_{10}$

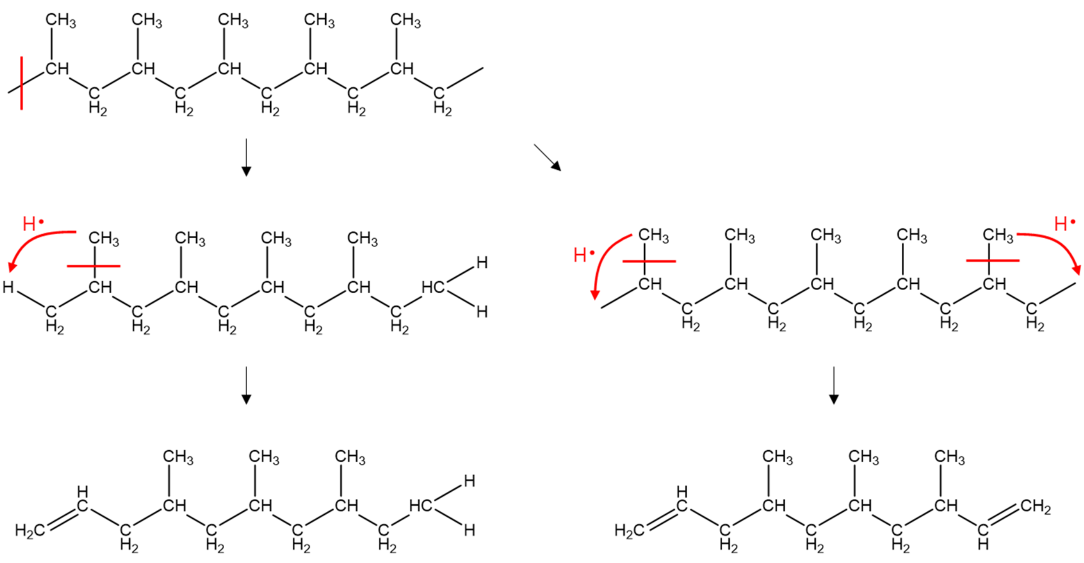

Figure S4. Partial pyrolysis pathway of PP proposed following the IR spectrum and TED-GC-MS

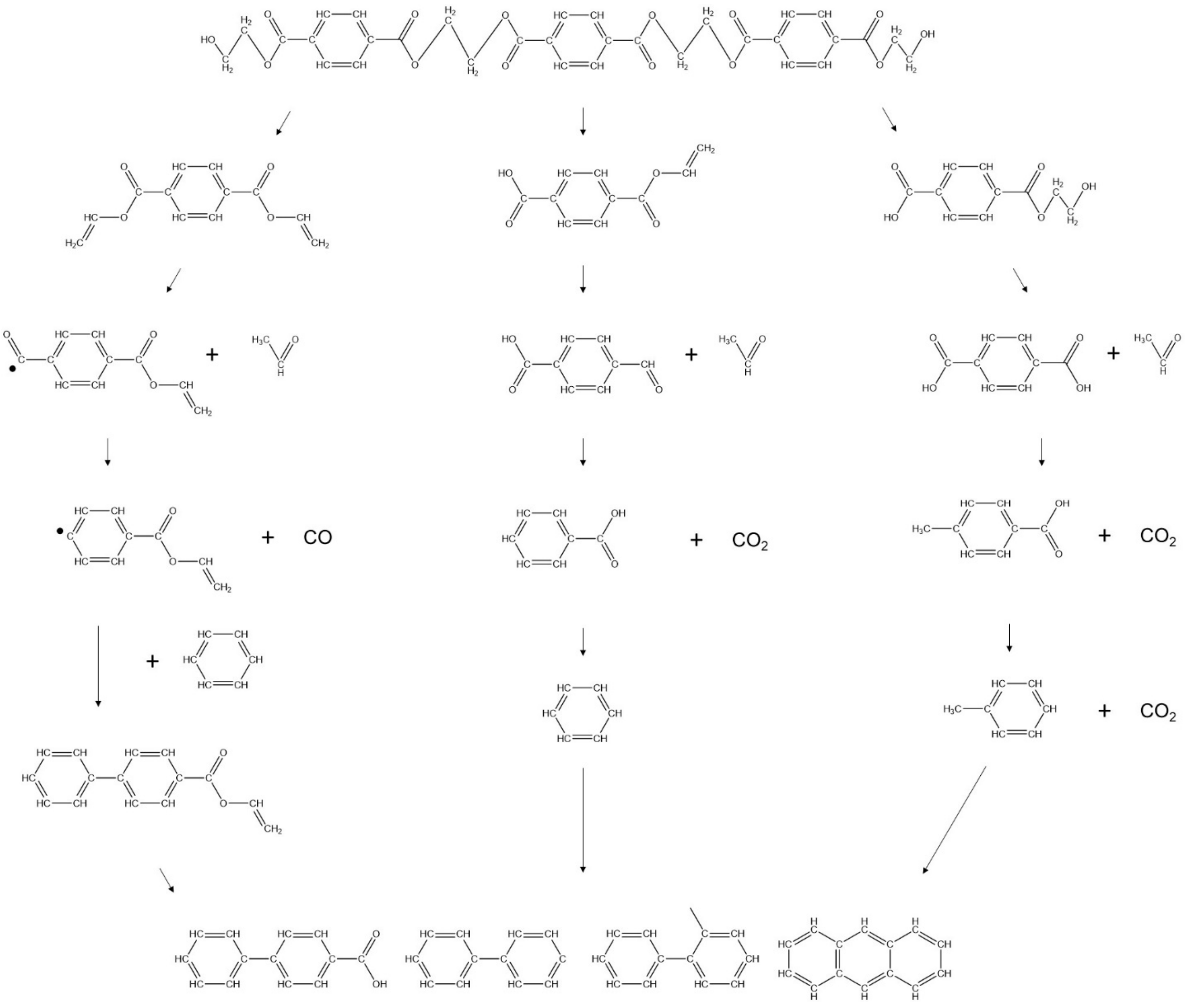

Figure S5. Pyrolysis pathway of PP proposed following the IR spectrum and TED-GC-MS, formation of some polycyclic aromatic hydrocarbons (PAH) may not be suggested by the IR spectrum and TED-GC-MS.

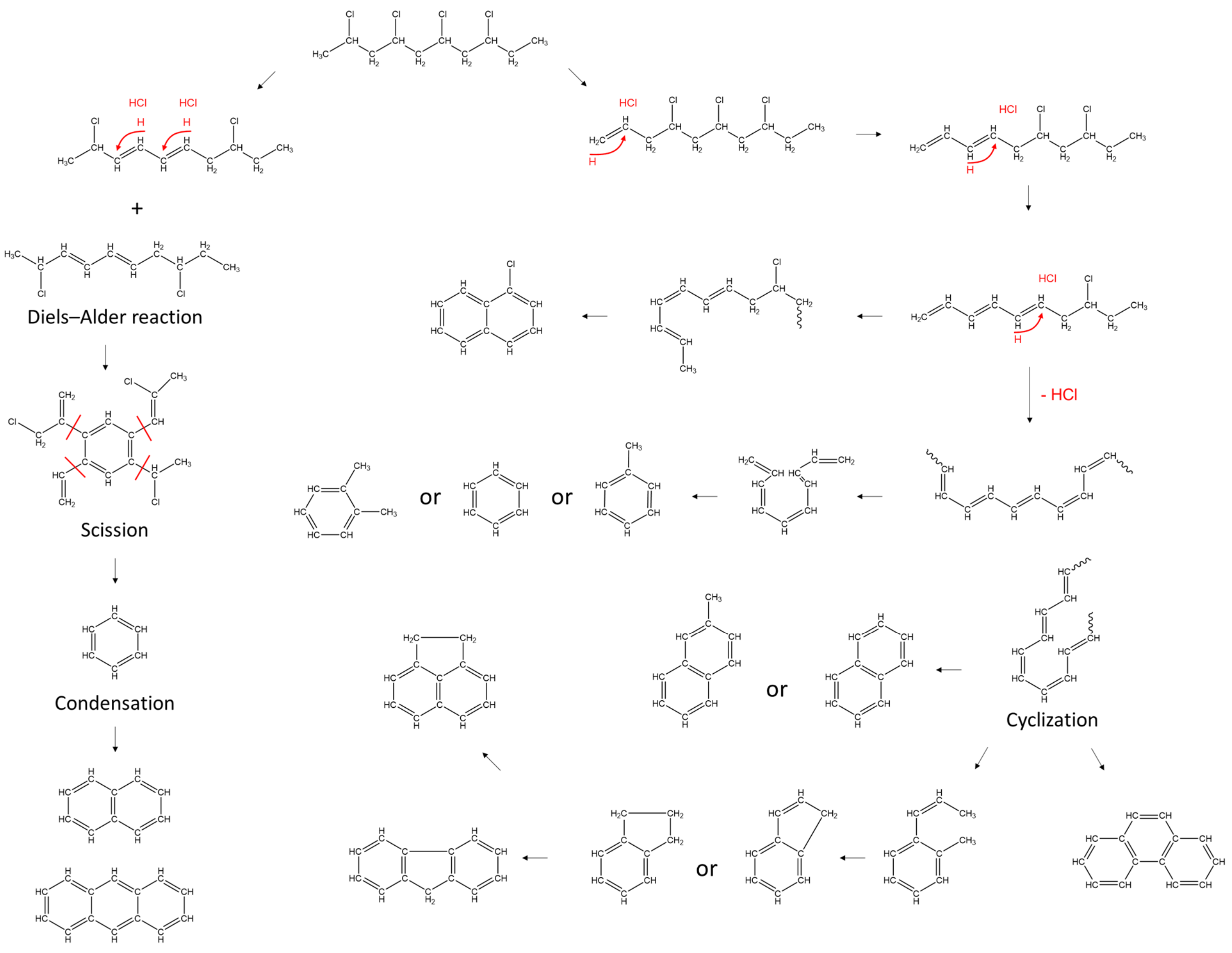

Figure S6. The thermal decomposition pathway of PVC suggested by IR spectrum and TED-GC-MS results
